# A Strategy toward Realizing Narrow Line with High Electrical Conductivity by Electrohydrodynamic Printing

**DOI:** 10.3390/membranes12020141

**Published:** 2022-01-24

**Authors:** Hongfu Liang, Rihui Yao, Guanguang Zhang, Xu Zhang, Zhihao Liang, Yuexin Yang, Honglong Ning, Jinyao Zhong, Tian Qiu, Junbiao Peng

**Affiliations:** 1 State Key Laboratory of Luminescent Materials and Devices, Institute of Polymer Optoelectronic Materials and Devices, South China University of Technology, Guangzhou 510640, China; 201530291429@mail.scut.edu.cn (H.L.); yaorihui@scut.edu.cn (R.Y.); msgg-zhang@mail.scut.edu.cn (G.Z.); 201921020680@mail.scut.edu.cn (X.Z.); 201530291443@scut.edu.cn (Z.L.); msyangyx@mail.scut.edu.cn (Y.Y.); 202010103138@mail.scut.edu.cn (J.Z.); psjbpeng@scut.edu.cn (J.P.); 2 Department of Intelligent Manufacturing, Wuyi University, Jiangmen 529020, China

**Keywords:** electrohydrodynamic jet printing, surface modification, annealing process, conductive

## Abstract

Over the past few decades, electrohydrodynamic (EHD) printing has proved to be an environmentally friendly, cost-effective and powerful tool in manufacturing electronic devices with a wire width of less than 50 μm. In particular, EHD printing is highly valued for the printing of ultrafine wire-width silver electrodes, which is important in manufacturing large-area, high-resolution micron-scale or even nanoscale structures. In this paper, we compare two methods of surface modification of glass substrate: UV treatment and oxygen plasma treatment. We found that oxygen plasma was better than UV treatment in terms of wettability and uniformity. Secondly, we optimized the annealing temperature parameter, and found that the conductivity of the electrode was the highest at 200 °C due to the smoothing silver electrode and the oxidation-free internal microstructure. Thirdly, we used EHD printing to fabricate silver electrodes on the glass substrate. Due to the decrease of conductivity as a result of the skin effect and the decrease of silver content, we found that driving voltage dropped, line width decreased, and the conductivity of silver line decreased. After the optimization of the EHD printing process, Ag electrode line width and conductivity reached 19.42 ± 0.24 μm and 6.01 × 10^6^ S/m, demonstrating the potential of electro-hydraulic printing in the manufacturing of flexible, wearable, high-density, low-power-consumption electronics.

## 1. Introduction

Microscale electrohydrodynamic printing (EHD) is a new micro/nano fabrication technology which has many advantages, such as low cost, simple structure, and direct forming without mask [1]. Due to these advantages, it is regarded as one of the best alternatives to the traditional lithography process. EHD printing shows great potential for industrial application in the preparation of solar cells [2], and in biomedicine [3], rollable electronics [4], micro/nano sensors [5], inverters [6], soft robots [7], and other micro/nano devices. At present, traditional inkjet printing has the advantages of simple equipment, low cost, simple operation, and low material consumption, but the accuracy of printing is still not high, which limits the development of super-resolution printed displays [8]. Compared with the inkjet printing technology, EHD printing has shown increased potential advantages in precision, material, cost, efficiency, and other aspects. EHD printing requires an electric field between the nozzle and the substrate, so that the ink at the nozzle is subjected to the electro-shear stress to form a Taylor cone. When the electric charge force is strong enough to overcome the liquid surface tension, the ink will spray out micro-droplets far smaller than the size of the nozzle [9,10]. More than a decade ago, Khan et al. used a multi-nozzle EHD inkjet print head for rapid printing, and realized the preparation of a 140 μm silver line, demonstrating the feasibility of EHD printing for the microelectronics industry [11]. Chen et al. used EHD printing to deposit particle-free silver inks on a polyimide substrate and electroless metal deposition to form conductive patterns. The final wire width of the copper electrode was 61 μm [12]. These studies indicate that EHD printing is an important method for the fabrication of micro/nanostructured devices. However, the resolution of these electronic microstructures has yet to be improved. EHD printing jet size is related to the diameter of the needle. High-resolution printing requires smaller needles, but smaller needles are more likely to clog. Therefore, this paper uses low-viscosity ink to avoid needle blockage.

Gold, silver, and copper inks are common printed inks for electronics. Silver nanoparticles are more economical than those composed of gold, and are more resistant to oxidation than copper. Because of their good thermal conductivity, electrical conductivity, solvent resistance, and high adhesion with most materials, silver nanoparticles have attracted extensive attention in many fields [13]. Many studies have focused on the direct printing of silver nanoparticles to produce high-resolution silver electrodes by using a low-viscosity ink [14,15,16]. However, ink needs surface treatment to improve spread on the substrate, thereby improving the uniformity of printing [17]. Here we optimize the surface uniformity, by surface treatment of the substrate, to avoid the diffusion of low-viscosity ink on the substrate to achieve reliable printing. Thermal annealing is an effective post-treatment technology. Y. J. Moon et al. studied the relationship between surface morphology and resistivity under different sintering temperatures and different thickness of silver nanoparticle ink layers [18]. This paper will discuss the effect of Ag/C ratio and cross-sectional area on conductivity after thermal annealing. In order to adapt to the development trend of printing electronics with high resolution, the goal of this study was to use EHD inkjet printing to minimize jet diameter and obtain high-resolution patterns while still having good electrical conductivity. Therefore, the experimental research and annealing process optimization carried out in this paper will provide directional guidance for improving the printing resolution and printing quality of EHD printing, and will lay the foundation and technical support for expanding the application of E-printing in the field of flexible electronics and wearable devices.

## 2. Materials and Methods

### 2.1. Materials

The substrate material chosen in this paper was glass. First, the substrate surfaces were cleaned ultrasonically in isopropyl alcohol and deionized water in sequence. This cleaning step was repeated twice. We used a commercial Ag nanoparticle ink (40TE-20C) which was purchased from Advanced Nano Products Co., Ltd. The ink solvent used was triethylene glycol methyl ether (TGME), and the mean particle size of silver nanoparticles was 80 nm. Before use, ink was treated ultrasonically for 10 min to avoid ink aggregation and needle clogging.

In order to study the influence of different surface treatments on the morphology of EHD printing process, the substrate was treated with UV and oxygen plasma. The UV treatment of surfaces was performed in an Ultraviolet curing apparatus (IntelliRay 400, West Springfield, Massachusetts, USA) with an output power of 60 W, for 10 min. When treated, the distance between the substrate and the lamp tube was 25 cm. The O_2_-plasma treatment of surfaces was performed in a multipurpose plasma system (Auto Glow 200, Tempe, Arizona, USA) with an output power of 60 W. During treatment, the chamber pressure was kept below 0.2 mTorr, and then at 0.5 mTorr with oxygen. Surface treatment was performed for 10 min.

### 2.2. EHD Printing for Silver Electrode

**Figure 1:**
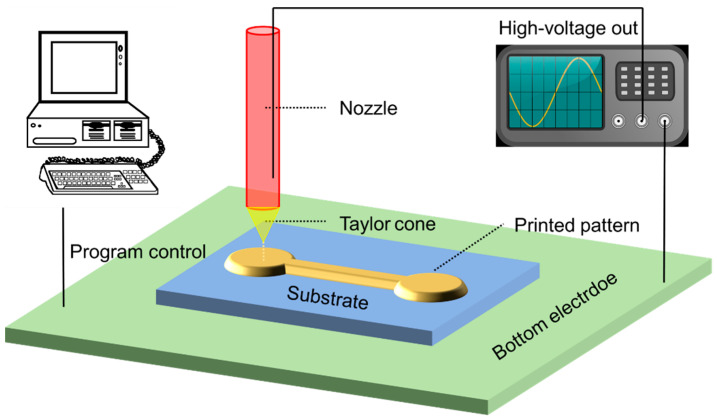
Schematic diagram of electrohydrodynamic (EHD) printing.

A commercial drop-on-demand (DOD) printer (SIJTechnology SIJ-S150, Tokodai, Ibaraki, Japan) was used to conduct the EHD printing. As shown in Figure 1, in EHD printing an electric field is applied between the needle and the substrate to make the ink squirt out. The needle was a quartz tube with a diameter of 2 μm. A 20 μL microloader (Eppendorf, Enfield, Hartford, USA) was used to load the ink into the needle. The printing linear array was processed with a driving voltage of 600 V and a printing speed of 20 mm/s. The driving voltage waveform was a square wave, the frequency was 1000 Hz, and the duty ratio was 75%. To study the effect of annealing temperature on conductivity, the Ag lines were annealed at 100 °C, 200 °C, 300 °C, or 400 °C for 5 min once they were jetted onto the glass substrate.

The influence of 540 V, 560 V, 580 V, 600 V, 620 V, 640 V, and 660 V printing voltages on the morphology and electrical conductivity of silver wires was studied by controlling the driving voltage then annealing at 200 °C for 5 min. 

### 2.3. Characterization

The contact angles of substrate were measured by optical contact angle measurement (Biolin Theta, Espoo, Helsinki, Finland). The surface morphology was investigated by scanning electron microscopy (Nova nanosem 430, Hillsboro, Oregon, USA) and confocal laser microscopy (Olympus OLS 5000, Shinjuku-ku, Tokyo, Japan). X-ray photoelectron spectroscopy (XPS) was carried out on a X-ray photoelectron spectrometer (ThermoFisher ESCALAB XI+, Pudong New Area, Shanghai, China). Energy-dispersive X-ray spectrometry (EDS) was used to analyze the change of the microscopic particle distribution and composition of the silver electrode film after annealing. To investigate the electrical performance, the electrical conductivity of a silver electrode based on EHD printing was measured using a semiconductor parameter tester (Primarius FS-Pro, Pudong New Area, Shanghai, China). The test voltage range was 0–3 V and the step size was 0.1 V. The calculation formula of conductivity is as follows:

σ = L/(S × R)(1)

where R is the volt–ampere characteristics measured by a semiconductor parameter instrument and obtained by linear fitting; L is the electrode length fixed at 1 mm; and S is the cross-sectional area of the electrode measured by a confocal laser microscope.

## 3. Results and Discussion

### 3.1. Surface Treatment

**Figure 2:**
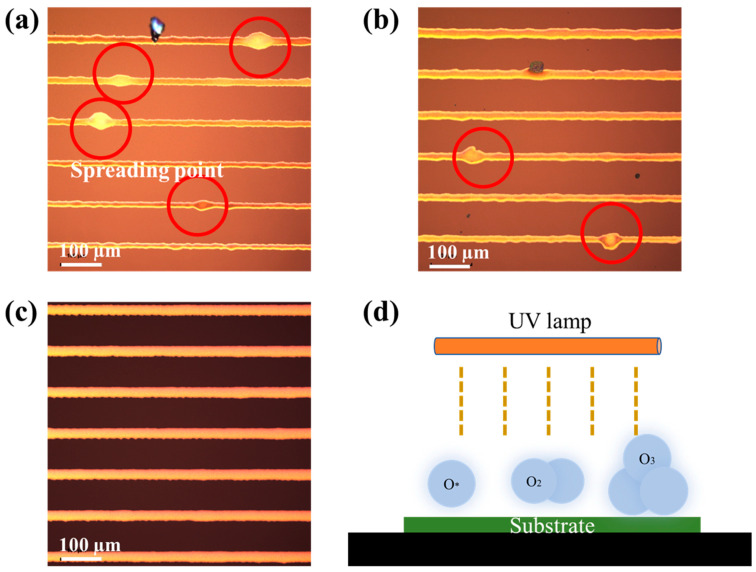
Microscopic pictures of the printed line: (**a**) untreated; (**b**) after UV treatment for 10 min; (**c**) after oxygen-plasma treatment for 10 min; (**d**) schematic diagram of oxygen-plasma treatment.

The relationships between surface treatment and processing time are shown in Figure S1. Surface treatment of glass substrates with ultraviolet light will change their physical and chemical properties [19]. For instance, UV irradiation will improve the wettability between the ink and the substrate because of decarbonization and the hydrophilic surface [20,21]. We can see from Figure S1 that with the increase of treatment time, both surface treatment methods could effectively reduce contact angle, and oxygen plasma was better than UV treatment in terms of wettability. Microscopic pictures of the printed line are shown in Figure 2. After surface treatment, silver lines could be directly printed on the substrates using EHD printing. The printability of the substrate without any treatment seemed to be the worst (Figure 2a) as silver ink diffused on the untreated substrate at random positions after printing. After UV treatment of the substrate, bulging points also appeared (Figure 2b). The substrate with oxygen plasma treatment had the best printing effect (Figure 2c). Oxygen plasma had better treatment results when compared with UV treatment, as it not only produced ultraviolet rays on the substrate, but also changed the substrate surface. With oxygen plasma treatment, a slight surface etch can be used for surface cleaning [22]. At the same time, the gas (O*, O_3_) activity in the chamber is high, which can effectively oxidize oil stains on the substrate surface (Figure 2d). Therefore, oxygen plasma treatment can reduce the unevenness of the substrate surface, thereby reducing the phenomenon of abnormal bulging. This approach could help to improve the uniformity of EHD printing.

### 3.2. Thermal Annealing

**Figure 3:**
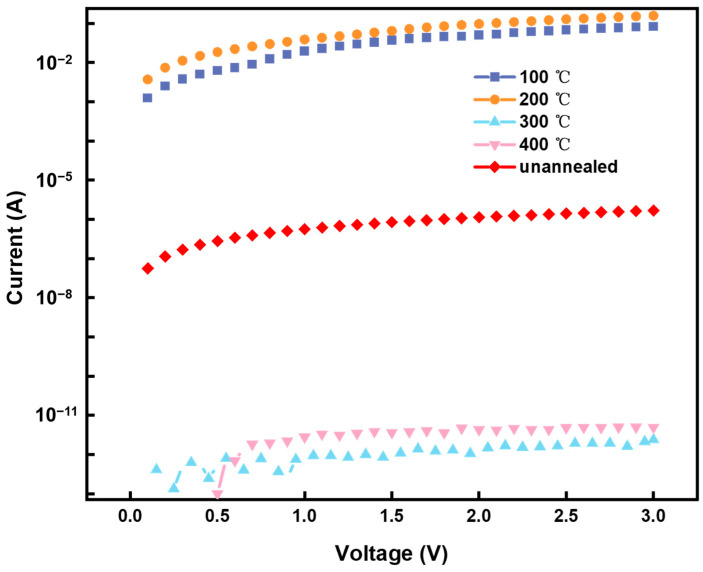
Volt−ampere curves under different thermal annealing conditions.

Figure 3 shows the relationship between annealing temperature and the conductivity of silver electrodes. Thermal annealing is an effective post-treatment technology that can be used for curing and sintering as-printed Ag electrodes [23,24]. As can be seen in Figure 3, the conductivity initially increased and then sharply decreased with increasing annealing temperature. From room temperature to 200 °C, the conductivity of the silver electrode was enhanced because the organic solvent evaporated, reducing the internal resistance. However, with the increase of annealing temperature, the internal defects of the electrode increased, resulting in a sharp decrease in conductivity. In order to prove that the internal defects led to the decrease of conductivity, the internal microstructure of the electrodes was observed with a scanning electron microscope.

**Figure 4:**
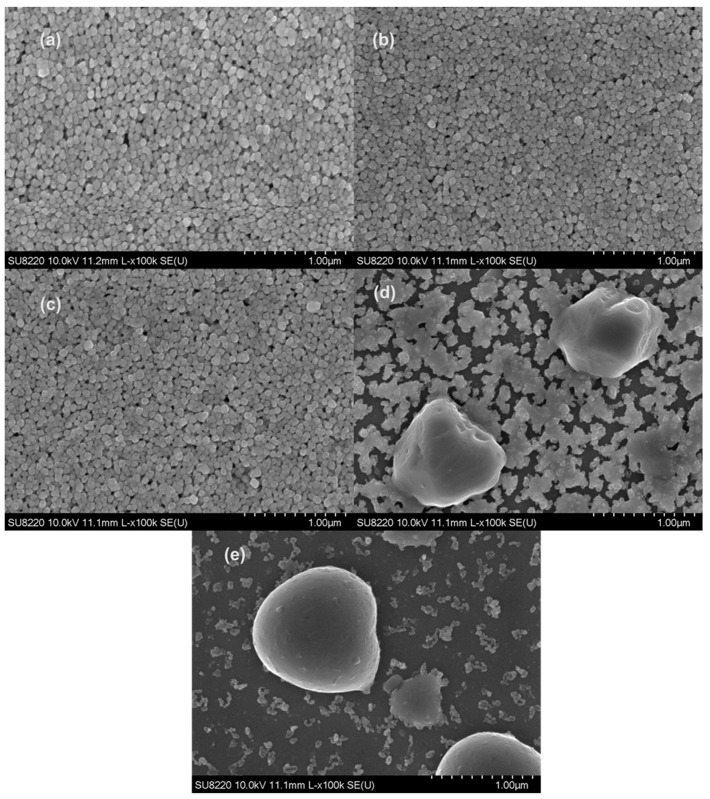
Silver electrode morphology under various annealing conditions: (**a**) as-printed; (**b**) 100 °C thermal annealing; (**c**) 200 °C thermal annealing; (**d**) 300 °C thermal annealing; (**e**) 400 °C thermal annealing.

Figure 4 shows the microstructure of particles inside the silver electrode at different annealing temperatures. The microstructure inside the silver electrode will eventually affect its conductivity. As can be seen in Figure 4, the size of silver particles under annealing at room temperature, 100 °C, and 200 °C was about 80 nm, and they had a spherical shape. Meanwhile, the distribution of Ag particles on the surface was uniform (Figure 4a–c). The silver particles were not tightly packed because many channels were formed by solvent evaporation. Though the melting point of bulk Ag is around 962 °C, we observed that silver particles melted and began to agglomerate to form large particles at only 300 °C (Figure 4d), which can be attributed to the size effect of nanoparticles [25,26]. When the annealing temperature was further increased to 400 °C, the Ag nanoparticles grew to isolated large-size Ag particles (Figure 4e). With the increase of annealing temperature, the silver particles inside electrode melted and fused into isolated particles, causing the breakage of conductive channels in the Ag electrode. Finally, the conductivity dropped sharply because there was an insufficient conductive percolation network.

**Figure 5:**
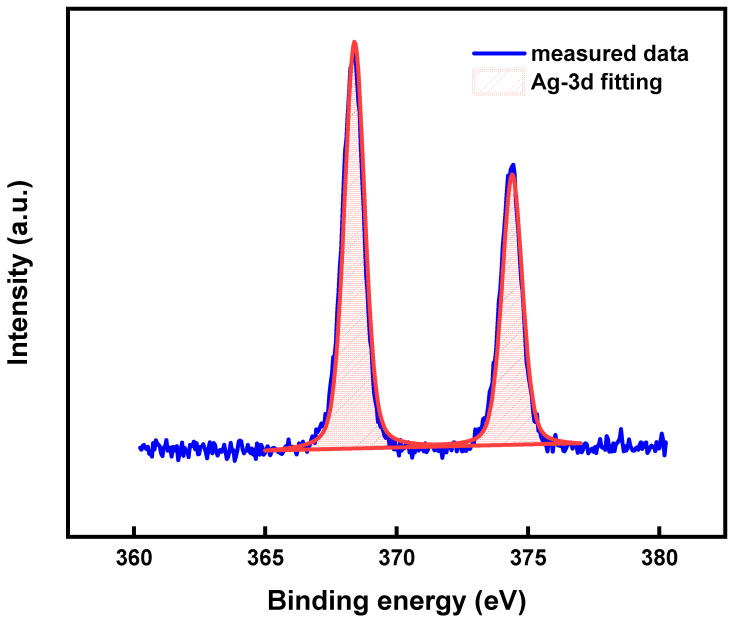
Ag-3d X-ray photoelectron spectroscopy (XPS) scanning atlas and fit analysis of the XPS spectra under 200 °C annealing.

Figure 5 shows the Ag-3d XPS scanning spectrum and XPS spectrum fitting analysis after annealing at 200 °C. Through the use of X-ray photoelectron spectroscopy (XPS), widely used both in basic research and in materials analysis (particularly in surface analysis), important information can be obtained about the electronic structure of Ag elements [27]. Figure 5 shows Ag-3d narrow-scan XPS spectra at 200 °C annealing. It was found that the Ag-3d peak shapes of the samples were symmetrical and sharp without passivation and disproportionation. Meanwhile, the spin-splitting orbits of Ag-3d_3/2_ and Ag-3d_5/2_ are roughly at 6 eV, and for pure silver the atomic splitting orbital is also 6 eV [28]. Therefore, we can preliminarily determine that the sample was in an elemental state. After the fine-scanning peak of Ag was calibrated with charge, we determined that the fitting effect was good by fitting with the standard peak of elemental silver at 368.40 eV [29]. The results indicate that Ag nanoparticles can remain in an elemental state without oxidation due to low-temperature annealing. Therefore, we recommend the annealing temperature of 200 °C among the investigated annealing temperatures, which can be used in the solidification molding of EHD printing silver particle ink and obtain good conductivity.

### 3.3. EHD Printing Ag Electrode Fabrication

**Figure 6:**
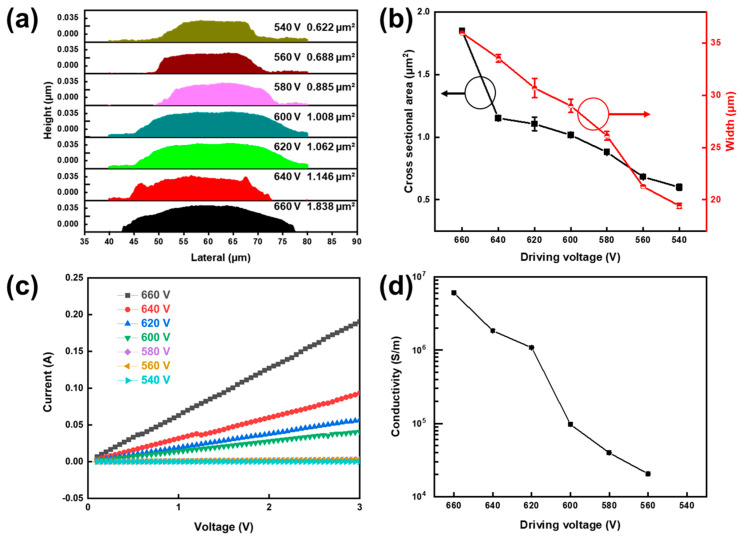
Profile morphology and electrical conductivity of EHD−printed silver electrodes: (**a**) Topography of cross−sectional area; (**b**) cross−sectional area and line width under different driving voltages; (**c**) volt–ampere curves under different driving voltages; (**d**) electrical conductivity under different driving voltages.

Figure 6 shows the morphology and conductivity of EHD-printed silver electrodes, the conductivity will be calculated by Formula 1. After O_2_-plasma surface treatment on the glass substrate, the silver rail was printed by changing the amplitude of the input driving voltage. Finally, the silver wire was annealed at 200 °C to complete annealing. The platform moving speed was 20 mm/s, and the pulse frequency was fixed at 1000 Hz. 

It can be seen from Figure 6a,b that the shapes of silver electrodes were thick in the middle and thin on both sides at different voltages. From 660 V to 540 V, it can be observed that the cross section of the silver electrode gradually shrank and a morphological transformation trend of both sides from protruding to concave can be observed. This was due to a reduction in the amount of ink jet. The relationship between the line width of printed silver and voltage amplitude is shown in Figure 6b. It can be clearly observed that the line width of the printed silver rail increased with the increase of amplitude. The maximum line width was 35.98 ± 0.01 μm and the narrowest was 19.42 ± 0.24 μm. Droplet size directly correlates with the dimension of the silver track on a substrate [30,31]. The larger the amplitude, the more charge accumulates on the meniscus and the larger the droplet size. Figure 6c shows the voltammetric characteristic curves under different printing conditions. For the EHD-printed silver electrode, the conductivity decreased monotonically with the decrease of the printing voltage. According to Ohm’s law, the resistance of silver electrodes can be obtained by linear fitting of voltammetric characteristic curves, so it can be said that the resistance of silver electrodes also decreased monotonically with the decrease of printing voltage. This is because the cross-sectional area of the silver electrode decreased, which reduced the current conduction path and therefore increased the resistance. 

It can be seen from Figure 6d that as the line width gradually decreased, the conductivity of the silver electrode decreased or even disappeared. The 660 V printed electrode had a conductivity of 6.01 × 10^6^ S/m. Compared with the former, the 560 V printed electrode had a higher resistance, with a conductivity of 2.04 × 10^4^ S/m We believe that the decrease of conductivity was due to the skin effect and the decrease of silver ratio.

**Figure 7:**
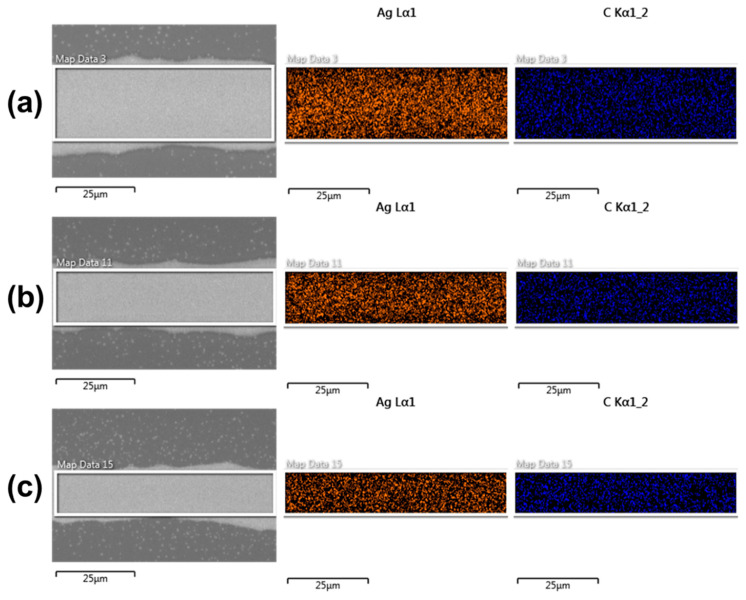
Scanning electron microscope (SEM) images and element distributions (Ag and C) scanned by energy-dispersive X-ray spectrometry (EDS): (**a**) 660 V; (**b**) 600 V; (**c**) 540 V.

Figure 7 shows EDS of silver and carbon under different driving voltages. EDS analysis of element content and distribution results show that the distribution of silver elements in the region was higher in the middle and less on the edge, which corresponds to the cross−sectional morphology of silver electrodes (Figure 7). Due to the skin effect, the current density reached a maximum near the edge of the silver electrode and decreased closer to the center [32]. The surface resistance was higher than the internal resistance because the density of silver particles on the cured surface was lower than the internal resistance. As the line width decreases, the proportion of surface resistance increases, so the conductivity decreases. The distribution of C element was relatively uniform and there was still some residual organic matter after annealing at 200 °C. Organic residue played the role of bonding silver nanoparticles and finally formed the surface morphology of silver electrodes.

**Figure 8:**
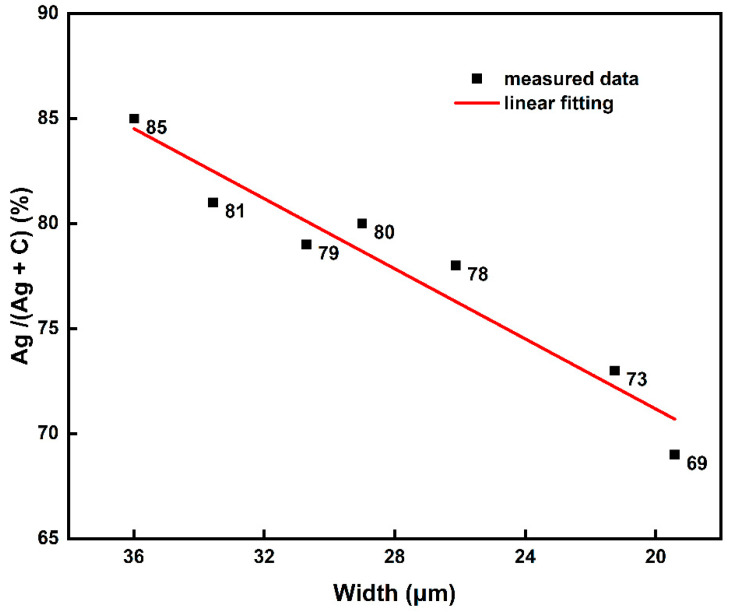
Relationship between silver/carbon content and line width.

The mass percentages of Ag and C under different printing conditions were calculated. According to the test results, the change of Ag/(Ag + C) under different line widths was solved (Figure 8). With the decrease of driving voltage, the inkjet quantity decreased. Ink particles have metal characteristics; silver nanoparticles have high dielectric constant and high electrostatic force, while polymers have low dielectric constant and small electrostatic force. Compared with the electrostatic force of the polymers, silver nanoparticles are more sensitive to electric field change. Therefore, when the driving voltage decreases, the Ag/C ratio will decrease. The decrease of Ag/(Ag + C) ratio indicates that the increase of organic residual ratio in the printed silver electrode increased the internal resistance of the electrode, causing the electrical conductivity of the silver electrode to decrease. In summary, high-voltage printing (with higher inkjet volume) yielded a larger line width and had higher conductivity, while low-voltage printing had a smaller line width and provided higher-resolution characteristics. This provides a reference for the preparation of EHD-printed electrodes with high performance at low temperature in the future, for example, increasing the number of silver particles by using an ink with higher solids content or reducing organic matter in the electrode by using other sintering methods.

## 4. Conclusions

We reported two methods for the surface modification of glass substrates. UV treatment and oxygen-plasma treatment were investigated to achieve reliable and high-uniformity Ag electrode EHD printing. We found that after oxygen-plasma treatment, bulging was avoided and the uniformity of EHD printing was improved. In addition, we optimized the internal microstructure of the electrodes by controlling the annealing process and realized the preparation of high-conductivity devices at low temperatures. We found that the best annealing temperature was 200 °C. This paper provides a reference for the preparation of EHD-printed silver nanoparticle electrodes with better comprehensive performance at low process temperatures in the future. Finally, we prepared a high-resolution silver electrode with a line width of 19.42 ± 0.24 μm and conductivity of 6.01 × 10^6^ S/m at low temperature through EHD printing, demonstrating the potential application of electrohydrodynamic printing in the manufacture of precision electronic devices.

## Data Availability

Data is contained within the article and supplementary materials.

## Supplementary Materials

The following are available online at https://www.mdpi.com/article/10.3390/membranes12020141/s1, Figure S1: Relationship between contact angle and treatment time.
