# Supplementary material for: A Strategy toward Realizing Narrow Line with High Electrical Conductivity by Electrohydrodynamic Printing
Source: Membranes (Basel). 2022 Jan 24;12(2):141. doi: 10.3390/membranes12020141 (PMC8879046; doi:10.3390/membranes12020141)
Supplement: Supplementary file 1 [file membranes-12-00141-s001.zip › membranes-1540281-supplementary.pdf]

membranes

Supplementary Materials

A Strategy toward Realizing Narrow Line with High Electrical Conductivity by Electrohydrodynamic Printing

Hongfu Liang [1], Rihui Yao [1], Guanguang Zhang [1], Xu Zhang [1], Zhihao Liang [1], Yuexin Yang [1], Honglong Ning [1,*], Jinyao Zhong [1], Tian Qiu [2,*] and Junbiao Peng [1]

1 State Key Laboratory of Luminescent Materials and Devices, Institute of Polymer Optoelectronic Materials and Devices, South China University of Technology, Guangzhou 510640, China; 201530291429@mail.scut.edu.cn (H.L.); yaorihui@scut.edu.cn (R.Y.); msgg-zhang@mail.scut.edu.cn (G.Z.); 201921020680@mail.scut.edu.cn (X.Z.); 201530291443@scut.edu.cn (Z.L.); msyangyx@mail.scut.edu.cn (Y.Y.); 202010103138@mail.scut.edu.cn (J.Z.); psjbpeng@scut.edu.cn (J.P.)
2 Department of Intelligent Manufacturing, Wuyi University, Jiangmen 529020, China
* Correspondence: ninghl@scut.edu.cn (H.N.); qiutian@ustc.edu (T.Q.); Tel.: +86-20-8711-4525 (H.N.)

Citation: Liang, H.; Yao, R.; Zhang, G.; Zhang, X.; Liang, Z.; Yang, Y.; Ning, H.; Zhong, J.; Qiu, T.; Peng, J. A Strategy toward Realizing Narrow Line with High Electrical Conductivity by Electrohydrodynamic Printing. *Membranes* **2022**, *12*, 141. https://doi.org/10.3390/membranes12020141

Academic Editor: Jasmina Casals Terre

Received: 20 December 2021
Accepted: 17 January 2022
Published: 24 January 2022

Publisher's Note: MDPI stays neutral with regard to jurisdictional claims in published maps and institutional affiliations.

Copyright: © 2022 by the authors. Submitted for possible open access publication under the terms and conditions of the Creative Commons Attribution (CC BY) license (https://creativecommons.org/licenses/by/4.0/).

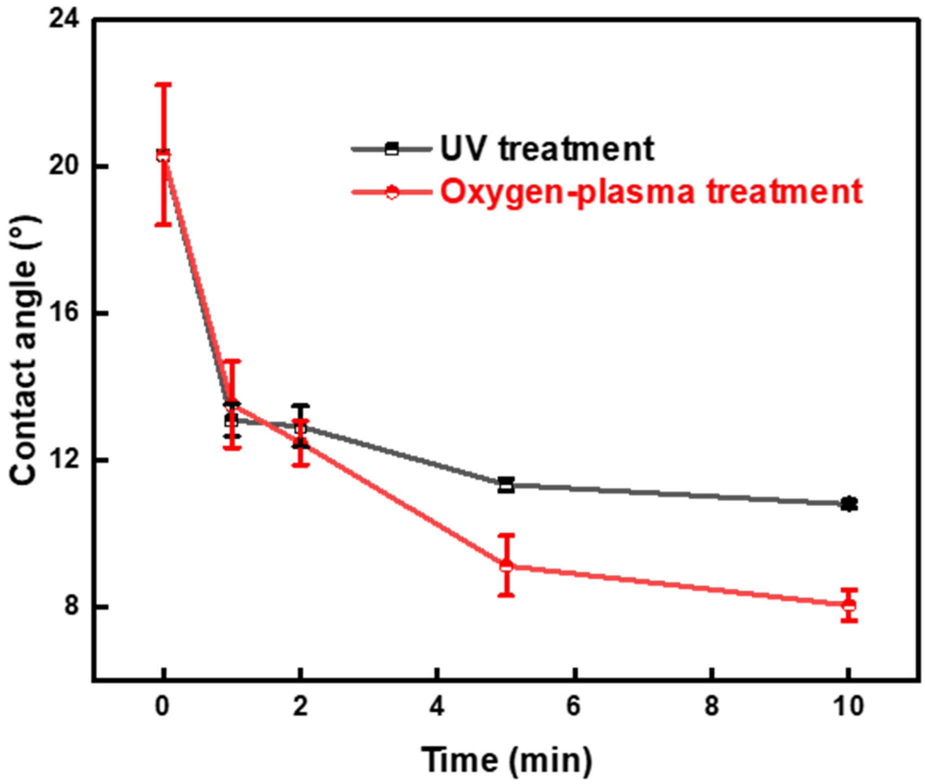

Figure S1. Relationship between contact angle and treatment time.
